# Full Transcriptome Analysis of Early Dorsoventral Patterning in Zebrafish

**DOI:** 10.1371/journal.pone.0070053

**Published:** 2013-07-29

**Authors:** Erika Fodor, Áron Zsigmond, Balázs Horváth, János Molnár, István Nagy, Gábor Tóth, Stephen W. Wilson, Máté Varga

**Affiliations:** 1 Department of Genetics, Eötvös Loránd University, Budapest, Hungary; 2 Institute of Biochemistry, Biological Research Centre of the Hungarian Academy of Sciences, Szeged, Hungary; 3 Biomi Ltd., Gödöllő, Hungary; 4 Agricultural Genomics and Bioinformatics Group, Agricultural Biotechnology Center, Gödöllő, Hungary; 5 Department of Cell and Developmental Biology, University College London, London, United Kingdom; University of Dayton, United States of America

## Abstract

Understanding the molecular interactions that lead to the establishment of the major body axes during embryogenesis is one of the main goals of developmental biology. Although the past two decades have revolutionized our knowledge about the genetic basis of these patterning processes, the list of genes involved in axis formation is unlikely to be complete. In order to identify new genes involved in the establishment of the dorsoventral (DV) axis during early stages of zebrafish embryonic development, we employed next generation sequencing for full transcriptome analysis of normal embryos and embryos lacking overt DV pattern. A combination of different statistical approaches yielded 41 differentially expressed candidate genes and we confirmed by *in situ* hybridization the early dorsal expression of 32 genes that are transcribed shortly after the onset of zygotic transcription. Although promoter analysis of the validated genes suggests no general enrichment for the binding sites of early acting transcription factors, most of these genes carry “bivalent” epigenetic histone modifications at the time when zygotic transcription is initiated, suggesting a “poised” transcriptional status. Our results reveal some new candidates of the dorsal gene regulatory network and suggest that a plurality of the earliest upregulated genes on the dorsal side have a role in the modulation of the canonical Wnt pathway.

## Introduction

The development of bilaterian embryos from a single-celled, fertilized egg into complex, multicellular, three-dimensional structure (the embryo) involves not only several rounds of cell divisions, but also a series of well-coordinated morphogenetic movements and patterning events, such as the establishment of the anteroposterior (AP) and dorsoventral (DV) axes.

Ever since the pioneering work of Hans Spemann and Hilde Mangold in the 1920s, it has been well known that during the formation of the DV-axis a specialized tissue with organizing properties is established in the future dorsal side of the embryo [1]. This tissue, called the Spemann organizer in amphibians, secretes signals that can instruct neighboring cells to form an axis. The molecular nature of these signals remained elusive for decades but with the introduction of molecular tools in developmental biology it became clear that several genes with organizing properties encode proteins that can antagonize BMP-signaling [2]–[7]. A parallel discovery of the “neural default model”, the property of BMP-signaling to suppress neural fate in ectodermal cells [8], [9] led to the recognition of the BMP-pathway as a major component of DV-axis patterning.

Zebrafish (*Danio rerio*) has become a widely used model organism, due to its fast development, high progeny number, transparency and ease of use. These advantages made it the subject of multiple extremely successful genetic screens, which have identified many key genes in diverse developmental processes, DV patterning being one of these [10]. From these screens, mutations that lead to DV patterning defects have often turned out to occur in genes that encode elements of the BMP signaling pathway (see [11] and references therein). However, some other key factors of early DV patterning have also been identified both by these screens and by other reverse genetic approaches [12]–[15]. The picture that emerges from these studies suggests that early zebrafish DV patterning is following a similar logic to that observed in the African clawed frog, *Xenopus laevis* [16].

A major similarity between the two anamniote model organisms is the pivotal early role of the canonical Wnt/β-catenin signaling pathway in dorsal determination. After fertilization, a dorsal determinant, Wnt11 in frogs [17], [18] and *wnt8* mRNA in fish [19], is transferred from the vegetal part of the oocyte to the future dorsal side. In zebrafish this process is mediated by an active, microtubule-dependent process [19]–[21] and results in the activation of canonical Wnt/β-catenin signaling on the presumptive dorsal side. Consequently, the dorsal transcriptional network is activated, which will ultimately lead to the expression of BMP-antagonists and the formation of the BMP-signaling gradient across the DV-axis. This activity gradient will have powerful patterning effects across the ectoderm and the mesoderm [22], [23].

The recessive, maternal-effect *ichabod* (*ich*) mutation causes severe ventralization due to an impairment in the early Wnt/β-catenin signaling pathway in embryos derived from mothers homozygous for the mutation (*ich* embryos) [24]. This defect is the result of the decreased accumulation of maternal *β-catenin-2* mRNA in the oocytes, caused most likely by a regulatory mutation [25].

Ventralized *ich* embryos lack a “shield” (the zebrafish equivalent of the Spemann organizer) during gastrulation and do not express any of the genes characteristic for organizer formation [24]. BMP-activity is also uniform across the ectoderm [26]. As the mutation can be completely rescued by the introduction of ectopic *β-catenin-2* mRNA, *ich* embryos provide an excellent paradigm to study the formation of the early organizer and to identify genes involved in this process. Using this experimental logic, previously we were able to discern the epistatic relationship between the Fgf-, Nodal- and BMP-signaling pathways [27].

In order to obtain a genome-wide view on the transcriptional changes related to the specification of the dorsal side in the early zebrafish embryos and to identify novel components of early DV patterning, we have sequenced full transcriptomes of untreated and rescued *ich* embryos at sphere stage (shortly after the midblastula transition – MBT –, the onset of zygotic transcription [28]) using RNA-Seq.

After mapping and analyzing one hundred million tags per sample and validating our results by *in situ* hybridization, we identified 32 genes differentially upregulated in rescued embryos, many of them implicated by prior studies in DV patterning and/or axial mesoderm formation. Our analysis shows that the largest group of activated genes are modulators of canonical Wnt-signaling. This suggests the existence of a robust self-regulatory network, to coordinate the dynamic changes in canonical Wnt activity during early stages of development.

## Results and Discussion

### Identification of Genes Expressed during Early Dorsal Induction using RNA-Seq

**Figure 1:**
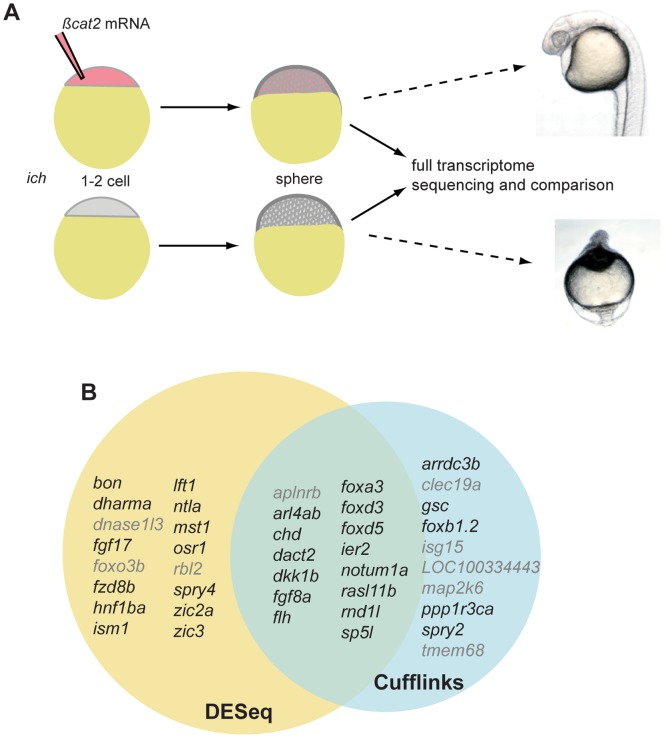
Experimental design and results of the comparative transcriptome analysis. (A) Total RNA samples were isolated from untreated (ventralized) *ich* embryos, and from “rescued” (*β-catenin* mRNA injected) counterparts. The samples were used for RNA-Seq, and the resulting datasets were compared to uncover genes upregulated during early dorsal specification. (B) Two independent *in silico* approaches were used to identify differentially expressed genes: DESeq and Cufflinks. The two scripts resulted in overlapping, but not identical gene-sets, which were combined for further analysis. (Grey demarks genes where differential dorsal expression could not be confirmed– see text for details.).

The ability to completely rescue the severely ventralized *ich* embryos created the prospect to analyze the early induction of the dorsal organizer in a completely unbiased manner using next generation sequencing methods. As the earliest molecular manifestation of the future dorsal organizer occurs around MBT, we decided to compare rescued and untreated embryos at this early stage, so we can identify the most upstream components of the dorsal induction network (Figure 1A). In order to avoid false positive hits arising from forced expression of Wnt-target genes, we injected *β-catenin-2* mRNA at concentrations that create overtly normal looking (non-dorsalized) embryos.

Using sequencing-by-ligation on the SOLiD V4 platform, we sequenced over one hundred million tags per sample. We first filtered and mapped the reads to the zebrafish genome (Zv9– Ensembl) using Genomics Workbench ver4.6. Next, in order to identify genes showing significantly altered expression level in “rescued” over untreated mutant (*ich*) sample we used a dual approach. On the one hand, we employed DESeq [29], a package developed to analyse count data from high-throughput sequencing (Figure S1A). For positive hits the alignments were manually analyzed in order to filter out false positive results. This conservative approach identified 32 genes, 17 of which have been implicated in organizer and/or axial mesoderm formation by previous studies.

In parallel we also used TopHat [30] splice junction mapper and Cufflinks [30], a package that assembles transcripts, estimates their abundances, and tests for differential expression in RNA-Seq samples (Figure S1B). After manual curation this approach resulted in 25 hits, with 12 of them previously known for their role in dorsal mesoderm formation.

The fact that established “dorsal” genes were overrepresented in our datasets underscored the strength of this approach and suggested that other genes on the list would prove to be *bona fide* components of the early DV patterning network.

The two datasets (DESeq and Cufflinks) only partially overlapped: just 15 genes were found in both lists (Figure 1B), with prominent dorsally-expressed genes missing from both (e.g. *gsc* in the case of DESeq and *dharma* for Cufflinks). As both datasets produced only conservative estimates, this was not surprising and showed the complementarity of the two approaches. Consequently for further analysis we used the combination of the two datasets.

To further test if our results consist of truly zygotic, early expressed genes, we compared our results against an independent dataset. Using data from a recent study of maternal-to-zygotic transition in zebrafish [31] (GEO database, accession number GSE22830), we found that most of the identified genes are indeed upregulated during MBT (Figure S2). Two genes, *map2k6* and *tmem68* had to be excluded from the combined dataset, as they showed, respectively, decreased and unchanged expression after MBT.

### Validation of the Results with *in situ* Hybridization

A further validation of our *in silico* dataset came from a series of *in situ* hybridization experiments, where we tested the expression pattern of the examined genes in both wild type and *ich* embryos at sphere stage and at 30% epiboly stage. In our previous studies we already performed such comparative expression studies for a small subset of key organizer-specific genes, such as *dharma*, *gsc* and *chd* [25]. We were able to obtain specific probes for 36 genes out of our 39 tested genes (the only exceptions being *arrdc3b*, *clec19a* and *rbl2*).

**Figure 2:**
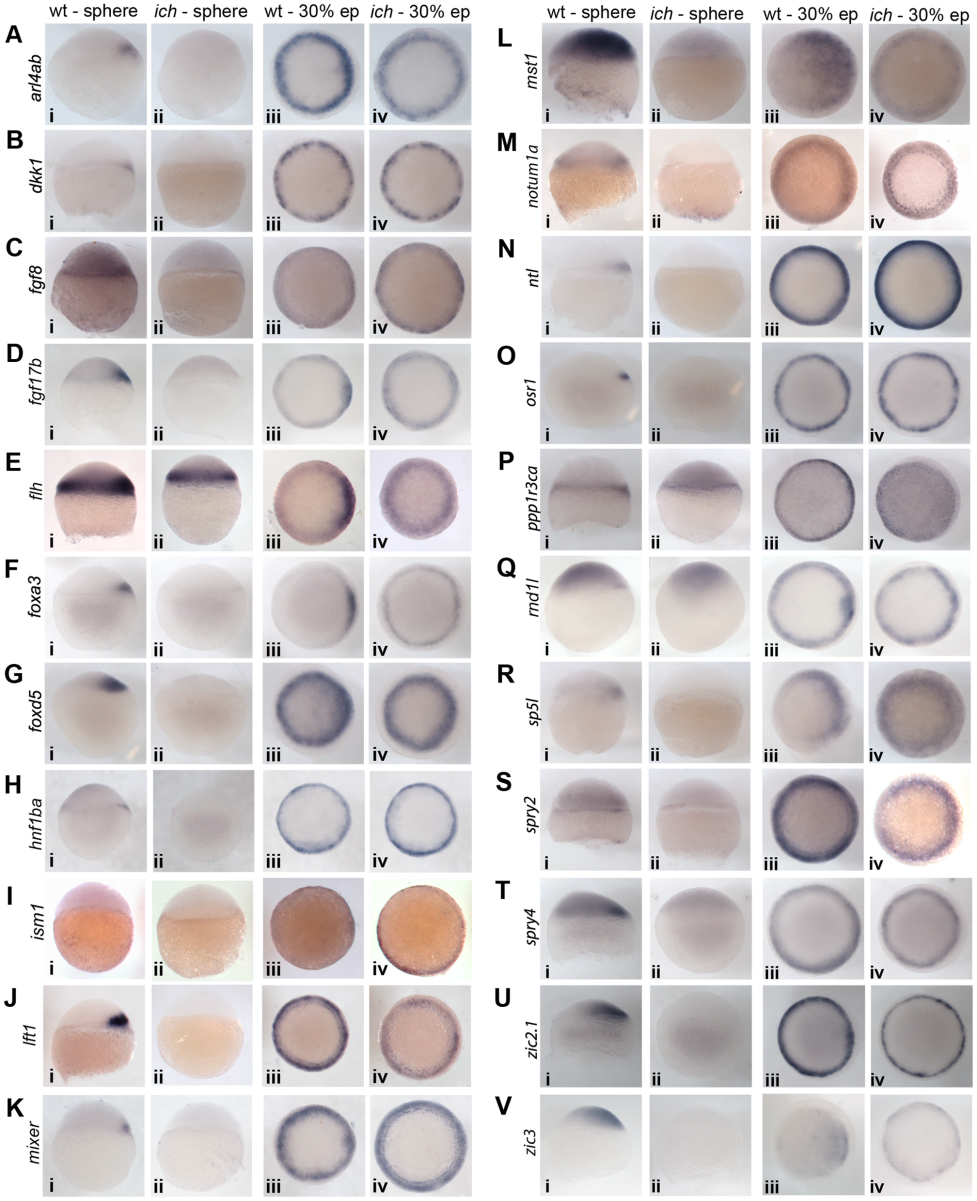
Expression of pan-mesodermal candidate genes. The majority of the identified genes show early dorsal expression in wild type embryos and lack thereof in *ich* controls. This is followed by a second wave of circumferential, mesodermal expression, which could be detected in both genetic backgrounds. An interesting exception is *flh*, which shows a somewhat counterintuitive expression dynamics: a very strong circumferential expression (complemented by strong dorsal signal in wild type embryos) can be detected as early as late sphere stage (Ei, Eii), but this expression becomes significantly weaker by 30% epiboly, except in the dorsal side of wild type embryos (Eiii, Eiv). Sphere stage embryos are shown from a lateral view, whereas ∼30% epiboly stage embryos are presented from an animal view (dorsal to the right in both cases).

**Figure 3:**
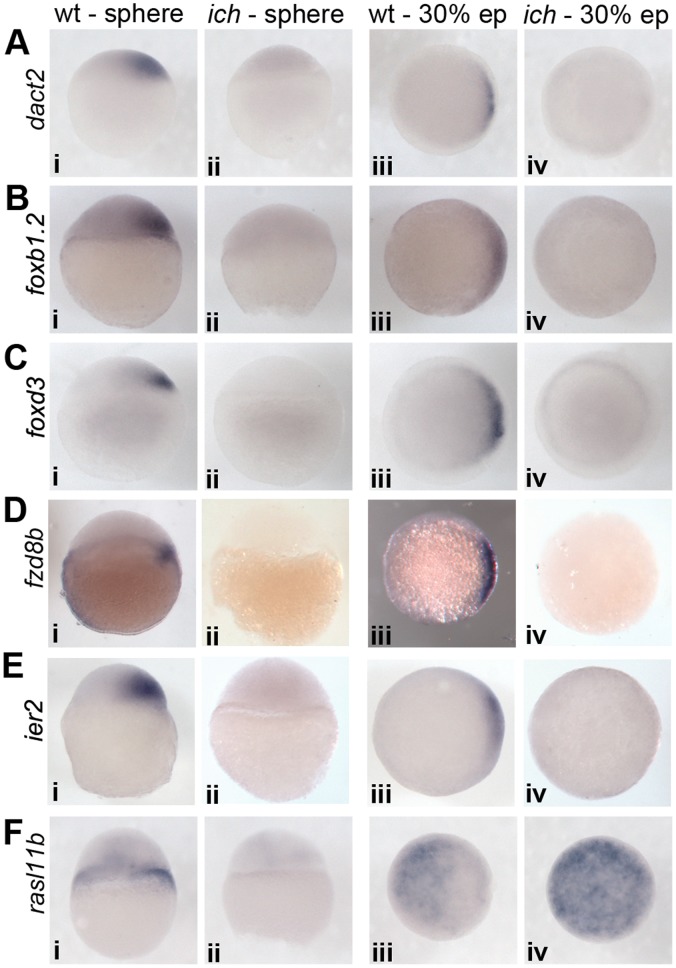
Expression of other candidate genes. The majority of genes in this category can be characterized by a “dorsal-only” expression pattern: they are expressed both at sphere and at 30% epiboly stages in the dorsal side of wild type controls and are absent from *ich* embryos. The exception is *rasl11b* which shows a strong dorsal, and a fainter ubiquitous expression at sphere stage in wild types (Fi), but by 30% epiboly it is absent from the dorsal side and expression can be detected only ventrally (Fiii). Accordingly, in *ich* embryos lacking dorsal induction, faint ubiquitous expression (Fii) becomes progressively stronger during development (Fiv). Sphere stage embryos are shown from a lateral view, whereas ∼30% epiboly stage embryos are presented from an animal view (dorsal to the right in both cases).

On the basis of the expression data, we observed two major phenotypic classes. Most genes showed early dorsal expression in wild type embryos, followed by circumferential upregulation in the germ ring at 30% epiboly stage (Figure 2). In *ich* embryos, while the early dorsal wave of expression was absent, the later circumferential expression was present (Figure 2). Other genes, similar to the dorsal-specific *gsc*, kept an “all dorsal” expression profile in wild type controls and showed restricted expression even at 30% epiboly (Figure 3). Such genes, as expected, were absent in *ich* embryos even at the onset of gastrulation. An interesting exception was *rasl11b*, which had an early dorsal expression domain (Figure 3Fi), but by mid-gastrula stages it was restricted to the ventral side of wild type embryos (Figure 3Fiii). In ventralized *ich* embryos, the early expression domain was missing (Figure 3Fii), while in later stage embryos the gene was expressed ubiquitously (Figure 3Fiv).

**Figure 4:**
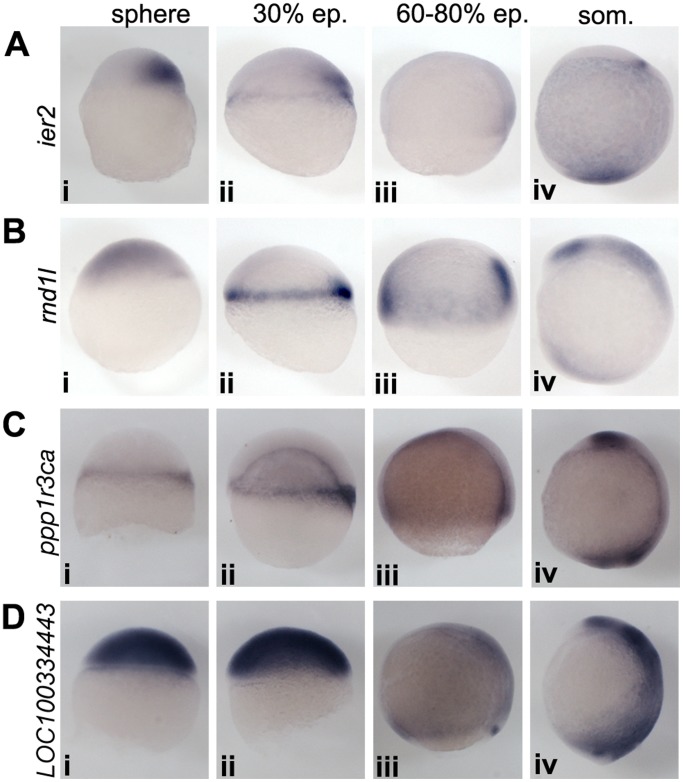
Expression patterns of newly characterized genes. (A) *ier2* expression starts early in the future dorsal side, persists until early mid-gastrulation, then fades away, and becomes detectable only during somitogenesis in a stripe in the hindbrain area, and in the tailbud. (B) *rnd1l* expression is weakly upregulated in all the blastomeres after MBT, and it is expressed at higher levels in a few dorsal precursors. Later the expression becomes restricted in the mesoderm (both axial and non-axial), where it is progressively downregulated. During somitogenesis two prominent neural expression domains appear: in the eyefield and in the hindbrain. (C) *ppp1r3ca* expression starts at the dorsal edge of the embryo, and later it is upregulated in a narrow stripe around the germring. Expression weakens during late gastrula stages, but it will be upregulated during somitogenesis in the anterior forebrain and in the posterior tailbud regions. (D) *LOC100334443* expression is strongly upregulated after the onset of zygotic transcription. During gastrulation it becomes restricted to the ectoderm, where it is gradually downregulated, except in the dorsal forerunner cells. During somitogenesis expression is also prominent in the neurectoderm, especially in the hindbrain. All embryos are shown from lateral view, dorsal to the right.

Two genes, *LOC100334443* and *dnase1l3* were upregulated only at MBT, but no significant difference between expression in wild type and *ich* embryos could be detected for either of them prior to late stages of gastrulation, when both were found to be specifically expressed in the dorsal forerunner cells (DFCs) of control embryos (Figure 4 and not shown).

Other genes, such as *aplnrb* and *isg15* completely lacked dorsal expression during the earliest stages of post-MBT development, and were present right after MBT in *ich* embryos, too. Both of these genes showed an apparently randomized expression before 30% epiboly, and later became restricted to the germring (Figure S3A-N and [32], [33]). As the expression pattern after MBT for these two genes ranged from a few cells to larger clusters, it is likely that the expression level differences detected by DESeq and Cufflinks were genuine, yet they reflected a stochastic difference only, thus these genes could be classified clearly as false positives.

We also observed *foxo3b* expression showing a similar, stochastic dynamic during blastula and gastrula stages (Figure S3O–H’). This is in contrast with the quasi-ubiquitous expression described before [34], however, repeated experiments yielded the same results. Of note, though expression seemed stochastic both in wild type and *ich* embryos, the overall expression appeared higher in wild types during early stages of development.

All three genes manifesting stochastic expression were previously associated with cellular and physiological stress [35]–[37], thus their collective post-MBT expression suggests that the blastomeres are particularly prone to stress at this stage.

### Promoter Analysis and Histone Modifications in Dorsal Genes

**Figure 5:**
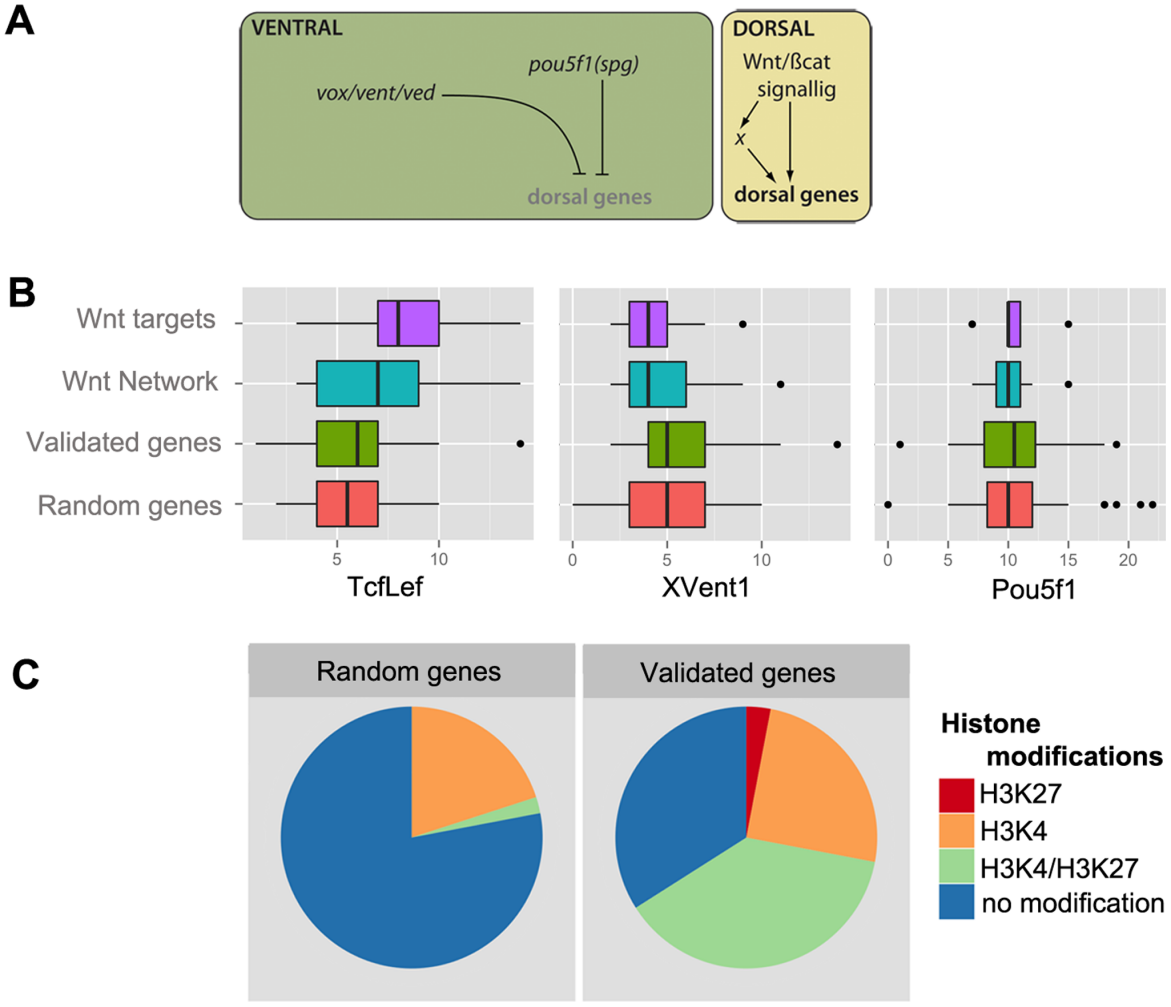
Promoter analysis and histone methylation patterns of candidate genes. (A) Previous work has identified the gene regulatory network (GRN) involved in the DV patterning of the zebrafish embryo. While in the dorsal side canonical Wnt signaling activates directly or indirectly the genes involved in the induction of the organizer, ventrally Vox/Vent/Ved and Pou5f1 represses the expression of these genes. (B) We tested the upstream 4 kb genomic region of the candidate genes for a possible enrichment in the binding sites of key transcription factors. There was no significant difference between the validated genes and a random gene set. (As expected, significant difference in the number of TcfLef binding sites could be detected between the promoter region of the confirmed Wnt-target subset of our gene-set, and the randomized set of genes (p<0.05).) (C) Using a previously published dataset we were able to demonstrate that the histones associated to our candidate genes often show a “bivalent status”, carrying activating (H3K4me3) and repressing (H3K27me3) methylation marks at the same time. This is in stark contrast with the histone-methylation patterns of randomly selected genes.

Previous work has established the core of the gene regulatory network (GRN) responsible for establishing the DV axis after the onset of the zygotic transcription [38]. While on the dorsal side canonical Wnt/β-catenin signaling, directly or indirectly, induces the expression of typical dorsal genes, on the ventral side maternal and then zygotic Pou5f1/Spg (the zebrafish homolog of mammalian Oct4), in combination with the homeobox repressors Vox, Vent and Ved inhibits the activation of the same dorsal genes (Figure 5A).

We wanted to test if the promoter regions of the candidate genes show enrichment for the binding sites of the aforementioned ventral transcription factors, or for recognition motifs of TcfLef, the transcriptional effectors of canonical Wnt-signaling. Therefore, using the Transcription Element Search System (TESS) script [39] we counted the number of such binding sites in the putative regulatory genomic regions of the analyzed genes. As transgenic reporter lines mimicking the expression of early transcribed developmental regulators often use 1–3 kb of the upstream genomic sequence [40]–[43], we decided to concentrate our efforts to the upstream 4 kb region.

Compared with a random set of zebrafish genes, we could not detect a statistically significant difference in the number of Pou5f1 and TcfLef binding sites (Figure 5B). When examining only the promoter regions of previously confirmed Wnt target genes in our gene-set, we did observe a significant enrichment in TcfLef sites, suggesting that our approach was sound. It appears then that the majority of dorsal genes detected by our transcriptome comparison are not direct targets of canonical Wnt-signaling. We also note that a tendency for enrichment for XVent1 binding sites can also be seen in the data, yet this difference was not large enough to be statistically significant (Figure 5B).

Of note, the enrichment of the TcfLef sites could not observed when we analyzed genomic regions that covered the first introns, or the −8 to −4 kb upstream regions or the downstream 4 kb genomic sequences of the validated and random genes (Figure S4). Interestingly, the analysis of the downstream 4 kb regions suggested a statistically significant difference in the number of Pou5f1 binding sites between the validated set and the random set of genes (Figure S4). This result, however, should be interpreted cautiously, as when the respective genomic regions of the validated genes known to be regulated by Pou5f1 [44] were compared with the rest of the validated dataset, no difference could be observed in the number of the Pou5f1 binding sites (not shown).

Recent results suggest that the nucleosomes at the promoters of developmentally important genes often acquire activating trimethylation marks on the lysine at position four (K4) in histone 3 (H3), or a “bivalent” mark of both activating K4 and inhibiting H3 lysine 27 (K27) trimethylation during transcriptional activation in zebrafish [45]–[47]. Using the recently published data of Lindeman and coworkers [46], we tested if our validated genes show similar patterns of epigenetic modifications.

Our validated “dorsal” gene set show an increase in the activating H3 K4 trimethylation marks in the promoter region, compared with the random set of genes, and an overabundance of “bivalent” histone-marks at the onset of zygotic transcription (Figure 5C), showing that dorsal genes are epigenetically marked prior to transcriptional activation. (Of note, according to the Lindeman et al. dataset [46] *dharma,* one of the earliest transcribed genes in the zebrafish genome [48], carries only H3K27 trimethylation during the time of its transcription. This could be an error in the dataset, but could also suggest a peculiar epigenetic regulatory mechanism at this genomic locus.)

### Many of the Dorsal Genes are Modulators of Wnt-signaling

We performed a manual literature search to assign biological function to the validated dataset. The results showed that the single biggest class of genes (11/32) was associated with modulation of Wnt-signaling, followed by regulators of cell motility (5/32) and modulators of Fgf-signaling (4/32) (Table 1).

**Table 1:** Known function(s) of confirmed candidate genes.

Gene name	Function	References
**Wnt-signaling modulators**		
*dharma*	Suppresses *wnt8* expression in the organizer precursor cells.	[74]
*dkk1b*	Extracellular Wnt-antagonist, which binds to Lrp6.	[60], [75], [76]
*foxa3*	Inhibits dorsal activation of *wnt8* transcription.	[77]
*mst1*	Tumor suppressor; homologue of *Drosophila hippo*.	[78], [79]
*foxd3*	Suppresses *dkk1* activation in the mesendoderm.	[80]
*fzd8b*	A putative receptor of Wnt-signaling.	
*gsc*	Homeodomain TF, which suppresses dorsal *wnt8* activation.	[77], [81]
*notum1a*	Inhibitor of canonical Wnt/signaling, which modifies glypicans.	[49], [82]
*ntla*	T-box trancription factor that regulates *wnt* gene expression.	[83]
*zic2a*	Inhibits transcriptional activation by β-catenin.	[84], [85]
*zic3*	Inhibits transcriptional activation by β-catenin.	[85]
**Fgf-signaling modulators**		
*fgf8a*	Fgf-receptor ligand important for dorsal- and isthmic organizers.	[27], [86]
*fgf17b*	Fgf-receptor ligand, with roles in early embryonic patterning.	[62]
*spry2*	Negative feedback modulator of MAPK activation.	[87], [88]
*spry4*	Negative feedback modulator of MAPK activation.	[87]
**Nodal-signaling modulators**		
*bon*	Associates to phosphorylated Smad2 to modulate Nodal signaling.	[89]
*lft1*	Feedback antagonist of Nodal signaling.	[90], [91]
**Regulation of cell motility**		
*arl4ab*	Small GTPase promoting actin cytoskeleton remodeling.	[92]
*dact2*	A modulator of the Wnt/PCP pathway that interacts with Dvl.	[93]
*ier2*	Involved in left-right patterning and CE movements.	[94], [95]
*rasl11b*	Small GTPase regulating mesendoderm development.	[96]
*rnd1l*	Rho family small GTPase modulating cell adhesion.	[97]
**Other**		
*chd*	Extracellular BMP antagonist.	[4], [98]
*flh*	Homeobox gene involved in notochord development.	[63]
*foxb1.2*	Forkhead family transcription factor.	[64]
*foxd5*	Specifier of neurectodermal fates.	[99]
*hnf1ba*	Regulates hindbrain and endocrine β-cell development idownstream of RA- and Fgf-signaling.	[100], [101]
*ism1*	Secreted angiogenesis inhibitor in mice.	[102]
*osr1*	STE20-kinase that regulates ion homeostasis.	[103]
*ppp1r3ca*	Regulatory subunit of protein phosphatase 1.	
*sp5l*	Transcription factor involved in mesendoderm and neurectoderm patterning.	[104]

**Figure 6:**
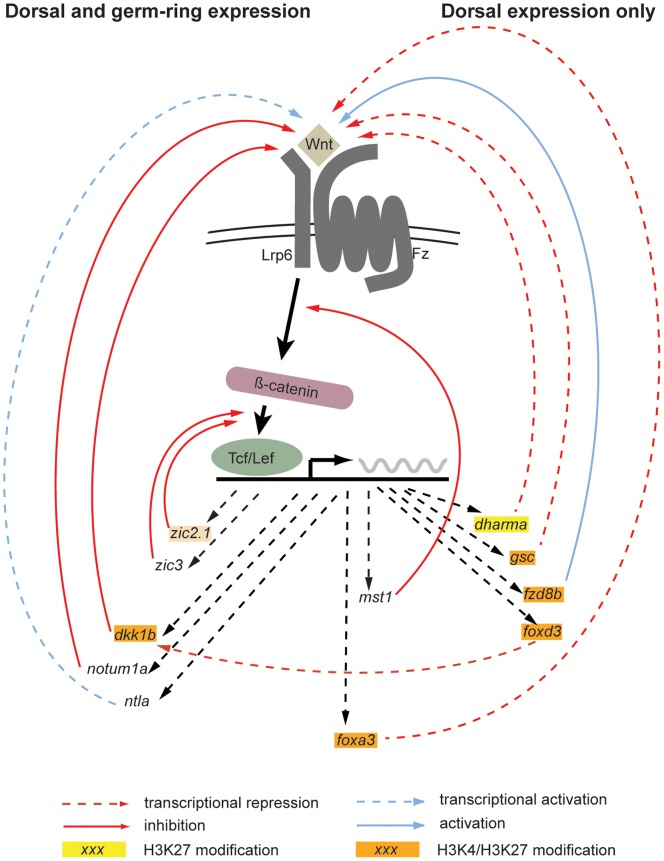
A plurality of early dorsal genes are modulators of the Wnt-signaling pathway. A close examination revealed that in our validated dataset genes that modulate Wnt-signaling are overrepresented. Interestingly, genes that are solely expressed in the dorsal side act usually as transcriptional repressors, while those genes that are also expressed in the ventrolateral mesodermal domains interact with the Wnt signaling pathway post-translationally. (Although *foxa3* and *mst1* both show circumferential expression, the dorsal expression domain is much more prominent for both genes, therefore we treated them as intermediates.) The most likely reason for this difference is that while dorsally Wnt-signaling has to be completely abolished to avoid its later ventralizing effects, in other domains the role of feedback repressors is just to ensure that Wnt-signaling is not overactivated. The majority of the Wnt-modulators carries “bivalent” H3K4/H3K27 histone trimethylation marks, suggesting they are in a “poised” status during MBT, which could also enhance the tight temporal and spatial control of the pathway. (In the case of *zic2.1* the “bivalent” status could be detected only prior MBT, and not during MBT, indicated by the lighter orange color.).

The enrichment in Wnt modulators is not surprising, as previous studies already demonstrated that several direct targets of Wnt-signaling are at the same time feed-back regulators of the pathway [49], [50]. Indeed, given the importance of Wnts during early zebrafish development a tight regulation can be expected. The close relationship between the pathway and its regulators is also underscored by an apparent enrichment in TcfLef binding sites in the promoter regions of the respective genes (Figure 5B). Furthermore, the “bivalent” epigenetic status observed at the majority of the Wnt-modulators (Figure 6) also suggests a tight and efficient regulation of this regulatory module.

Regulation of Wnt-signaling can happen at the transcriptional level (by inhibiting/activating the expression of specific components of the canonical pathway) or at the translational level (by modulating the function of proteins). Interestingly, Wnt-modulators associated with transcriptional repression showed an elevated dorsal (*foxa3*) or dorsal-only (*dharma, gsc*) expression pattern, while the majority of post-translational repressors (*dkk1b, notum1a, zic2.1, zic3*) were upregulated circumferentially in the germ-ring at the onset of gastrulation (Figure 6).

These differences can be explained in the context of the changing role of Wnt-signaling during early development. While the early activation of the canonical Wnt/β-catenin pathway is indispensable for the formation of the organizer and the specification of dorsal cell fates, at the onset of gastrulation Wnt (specifically Wnt8) becomes a potent morphogen with ventralizing and posteriorizing effects [11]. Due to this later role, pathway activation has to be completely excluded from the dorsal side.

The second wave of Wnt-signaling is driven by the activation of *wnt8* in the germring, by Nodal-signaling emanating from the yolk syncitial layer (YSL) [51]–[53]. As this signal is circumferential, dorsal transcriptional repressors counteract its effects by inhibiting the activation of *wnt8* in this region.

When Wnt-signaling becomes a potent posteriorizer during gastrulation, its levels have to be tightly regulated. Too much Wnt activity results in the expansion of posterior tissues at the expense of anterior ones, while reduced Wnt-signaling has the opposite effect. To achieve such a fine-tuning of Wnt activity, post-translational modulation is an ideal tool. While not interfering with the source of Wnt-signaling, the circumferentially expressed Wnt-suppressors can set the level of Wnt activity through well characterized negative feed-back mechanisms.

### Conclusions

Using an unbiased full transcriptome sequencing approach we generated a comprehensive list of post-MBT dorsally induced genes in the zebrafish embryo (Figure 1B). Our results suggest that the majority of these genes are epigenetically marked during the activation of zygotic transcription, often with “bivalent” H3 K4/K27 trimethylation (Figure 5C). Given that in *Xenopus* dorsal β-catenin has a role in establishing a “poised“ chromatin state prior to MBT [54], we propose that *ich* embryos, due to insufficient β-catenin-2 levels [25], lack these chromatin modifications and consequently fail to induce their dorsal developmental programs.

We also show that many of the dorsally activated genes are modulators of the Wnt-pathway, but components of the Fgf- and Nodal-signaling pathways are also induced together with genes important for cell motility and adhesion (Table 1).

Of note is the absence of *sqt/ndr1* from our list, as it was previously shown to be an early essential component of the dorsal induction pathway [27]. This finding is most likely explained by the maternal component of *ndr1*, also observed in *ich* embryos [55], wich will buffer the changes occurring during MBT at the dorsal side. The activation of the Fgf-signaling pathway, itself dependent on Nodal-signaling [27] is also indicative that the dorsal activation of *ndr1* in fact occurs.

It is also noteworthy that several genes on our list are not direct targets of Wnt-signaling. As mentioned above, the transcription of Fgf ligands requires prior activation of Nodal-signaling. Furthermore, other genes, such as the Fgf-signaling feedback inhibitors *spry2* and *spry4*, and the BMP-antagonist *chd* are dependent on the activation of Fgf-pathway [27], [56], [57]. Taken together, this suggests that several levels of the dorsal GRN are activated promptly and quickly after MBT.

Finally, although most of the examined genes showed an early dorsal activity, followed by a later, pan-mesodermal expression in the germ-ring, a couple of genes were restricted to the dorsal side. It will be interesting to understand the transcriptional logic regulating the expression of the latter genes, as it might reveal how context-dependent gene activation can be encoded on genomic level.

Our analysis also determined the expression pattern during early development for several, previously uncharacterized genes, such as *ier2, rnd1l, ppp1r3ca* and *LOC100334443* (Figure 4). Although not all of them show restricted dorsal expression during blastula and early gastrula stages, for those that do, it will be interesting to determine how they fit into the established genetic network of dorsal induction in zebrafish embryos.

## Materials and Methods

### Fish Care

Wild-type *ekwill* (*ekw*) and mutant *ich^p1/p1^* fish stocks were maintained in the animal facility of Eötvös Loránd University. All protocols used in this study were approved by the Hungarian National Food Chain Safety Office (Permit Number: XIV-I-001/515-4/2012).

### RNA Preparation

Rescue experiments were performed as described before [27]. Total RNA was isolated from 50–50 uninjected and *β-catenin-2* mRNA injected embryos with TRIZOL (Invitrogen), using the manufacturer’s protocol. An extra round of ethanol precipitation was applied in order to eliminate residual TRIZOL contaminations.

### Whole Transcriptome Sequencing

RNA quality and quantity measurements were performed on Bioanalyzer (Agilent Technologies) and Qubit (Life Technologies). High quality (RIN >8.5) total RNA samples from three biological replicates were pooled and processed using the SOLiD total RNA-Seq Kit (Life Technologies), according to the manufacturers suggestions. Briefly, 5 µg of pooled RNA was DNaseI treated and the ribosomal RNA depleted using Eukaryote RiboMinus rRNA Removal Kit (Life Technologies). The leftover was fragmented using RNaseIII, the 50–200 nt fraction size-selected, sequencing adaptors ligated and the templates reverse transcribed using ArrayScript RT. The cDNA library was purified with Qiagen MinElute PCR Purification Kit (Qiagen) and size-selected on a 6% TBE-Urea denaturing polyacrylamide gel. The 150–250 nt cDNA fraction was amplified using AmpliTaq polymerase and purified by AmPureXP Beads (Agencourt). Concentration of each library was determined using the SOLiD Library TaqMan Quantitation Kit (Life Technologies). Each library was clonally amplified on SOLiD P1 DNA Beads by emulsion PCR (ePCR). Emulsions were broken with butanol, and ePCR beads enriched for template-positive beads by hybridization with magnetic enrichment beads. Template-enriched beads were extended at the 3′ end in the presence of terminal transferase and 3′ bead linker. Beads with the clonally amplified DNA were deposited onto sequencing slide and sequenced on SOLiD V4 Instrument using the 50-base sequencing chemistry.

### Data Availability

Short-read data of the two sequenced transcriptomes were deposited in NCBI’s Short Read Archive at http://www.ncbi.nlm.nih.gov/sra/under accession SRA075737.

### Bioinformatics

Bioinformatic analysis of the whole transcriptome sequencing was performed in color space using Genomics Workbench ver4.6 (CLC Bio). Raw sequencing data were trimmed by removal of low quality, short sequences so that only 50 nucleotide long sequences were used in further analysis. Sequences were mapped in a strand specific way onto the *Danio rerio* Zv9 (Ensembl) reference genome, using default parameters. In order to avoid possible false positive hits, genes where the mapped reads showed a highly skewed distribution in the “rescued” dataset were removed from further analysis. DESeq results were manually curated, to remove (further) false positive hits, which showed highly skewed mapping of reads. Only genes where a 1.5 fold upregulation was detected after normalization were considered for further analysis.

As an alternative approach, the 50 nucleotide long color-space RNA-Seq data were mapped onto the *Danio rerio* Zv9 reference using TopHat v1.3.2 [58], which, by allowing for recognition of splice junctions, facilitates correct mapping of sequencing reads that span multiple exons. Assembly of transcripts and estimation of their relative abundance between the two samples was carried out using Cufflinks v1.1.0 [59]. For candidate genes the alignments in the rescued dataset were manually checked to avoid false positive arising from uneven distribution of reads. (In a typical false positive sample more than half of the reads mapped to a short, 20–30 bp long segment.).

For promoter analysis the indicated genomic sequences were downloaded from Ensembl both for our validated gene set and a randomized set of genes, and analyzed using TESS (http://www.cbil.upenn.edu/tess) [39] for TcfLef, XVent1 and Pou5f1 binding sites. Only hits with a log-odd score better than 8.0 were counted. The number of such binding sites for each gene was recorded (Table S1) and analyzed with the R program package (http://www.r-project.org).

Histone modification status for the validated and random genes was determined using the dataset of Lindeman and coworkers [46]. Data visualization was performed with the ggplot2 package (http://ggplot2.org).

The random gene-set was generated using the corresponding application of the RSA-tools package (http://rsat.ulb.ac.be/rsat/random-genes_form.cgi) and can be found in Table S1.

### *In situ* Hybridization

Whole mount *in situ* hybridization stainings were performed as described before [25]. The plasmids used for probe synthesis were as follows: pCS2+aplnrb [32], pZL-dkk1 [60], pCS2+fgf8 [61], pcDNA3.0-fgf17b [62], pBS-flh [63], pBS-foxa3 [64], pBS-foxb1.2 [64], pBS-foxd3 [64], pGEMT-foxd5 [65], pSPORT1-ism1 [66], pBS-lft1 [67], pGEMT-mixer [68], pBS-ntl [69], osr1 [70], pCRII-TOPO-sp5l [66], pBS-zspry2 [71], pBS-spry4 [56], pBS-zic2.1 [72], zic3 [73]. The remainder genes were cloned through nested PCRs and cloned into pCRII-TOPO-Blunt (Invitrogen) or pGEM-T-Easy (Promega) vectors. PCR primers were designed based on the available annotations in the Ensembl database, and their sequences can be found in the Methods S1 file.

## Supporting Information

Figure S1 **Identification of candidate genes.** Genes showing a significant (more than 1.5 fold in the case of DESeq) upregulation after normalization were considered for further analysis. Two complementary approaches, DESeq ([29] in the Main Text) (A) and Cufflinks ([30] in the Main Text) (B) packages were used to identify differentially regulated genes. Positive hits are colored red.
(TIF)

Figure S2 **Majority of the candidate genes are upregulated after MBT.** Using a previously published dataset ([31] in Main Text), we tested whether the expression of our candidate genes is upregulated at MBT, as expected. Our results show that indeed, this is the case for all, except two genes: *map2k6* and *tmem68*. The former showed a weak maternal expression, which was downregulated after MBT, whereas the latter showed no change in expression during and after MBT.
(TIF)

Figure S3 **Genes with stochastic early expression.** Genes belonging to this class show stochastic expression at the onset of the zygotic transcription. We could detect clusters of cells both in wild type and *ich* embryos that upregulated these particular genes, but no clear pattern emerged. For *aplnrb* (A–F) and *isg15* (G–N), the expression pattern later became more coherent, and localized around the germring. Interestingly, although no clear expression pattern emerged, *foxo3b* expression in general appeared higher in wild type embryos (O–V). Later stages of *foxo3b* expression in wild type embryos also showed stochastic and varying expression patterns (A’–H’). All sphere and 30% epiboly stage embryos are shown from an animal view. Shield, 70% epiboly and bud stage embryos are shown from a lateral view, with dorsal to the right.
(TIF)

Figure S4 **Extended genomic region analysis of validated genes.** (A) For multi-exon genes, statistical analysis of the first introns for enrichment in putative TcfLef, XVent1 or Pou5f1 binding sites yielded negative results. (B) In the 4 kb downstream genomic regions, we could not detect significant differences between the random and validated gene-sets in the number of TcfLef and XVent1 sites. The validated gene-set was enriched however in putative Pou5f1 sites (p<0.05). However, this result has to be interpreted with caution, as proven Pou5f1-targets [44] within the validated set have about the same number of potential Pou5f1 binding sites as non-target counterparts (not shown). (C) Analysing the −8 kb to −4 kb upstream genomc region no differences were observed in the number of TcfLef, XVent1 and Pou5f1 sites between our validated and random data-sets.
(TIF)

Table S1 ***In silico*** **binding site analysis for candidate and randomly selected genes.**
(XLS)

Methods S1 **Sequence of PCR primers used for the nested PCR reactions.**
(DOC)
