# Supplementary figures and images for: Full Transcriptome Analysis of Early Dorsoventral Patterning in Zebrafish
Source: PLoS One. 2013 Jul 29;8(7):e70053. doi: 10.1371/journal.pone.0070053 (PMC3726443; doi:10.1371/journal.pone.0070053)

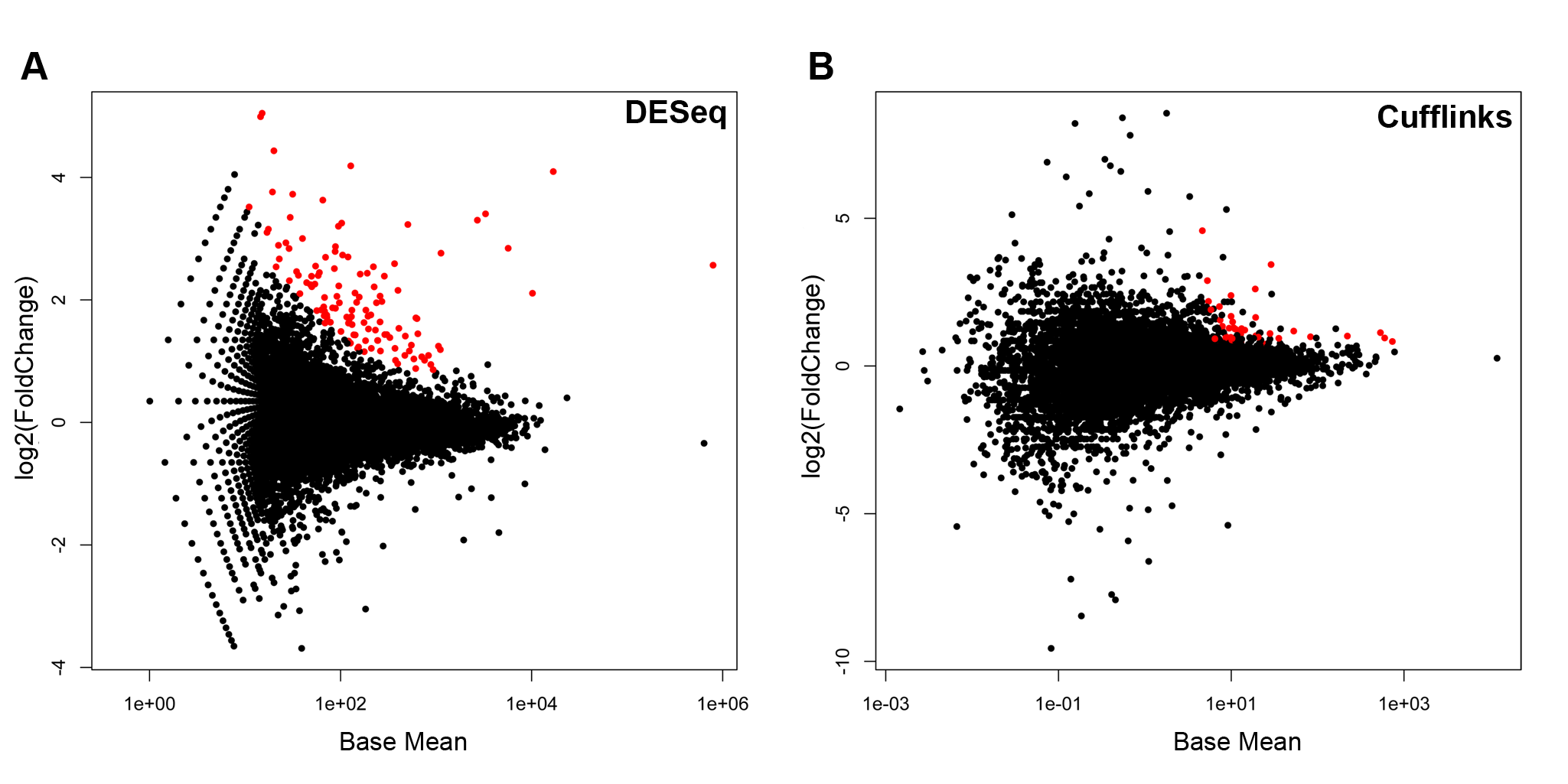

Supplement: Figure S1 — Identification of candidate genes. Genes showing a significant (more than 1.5 fold in the case of DESeq) upregulation after normalization were considered for further analysis. Two complementary approaches, DESeq ([29] in the Main Text) (A) and Cufflinks ([30] in the Main Text) (B) packages were used to identify differentially regulated genes. Positive hits are colored red. (TIF) [file pone.0070053.s001.tif]

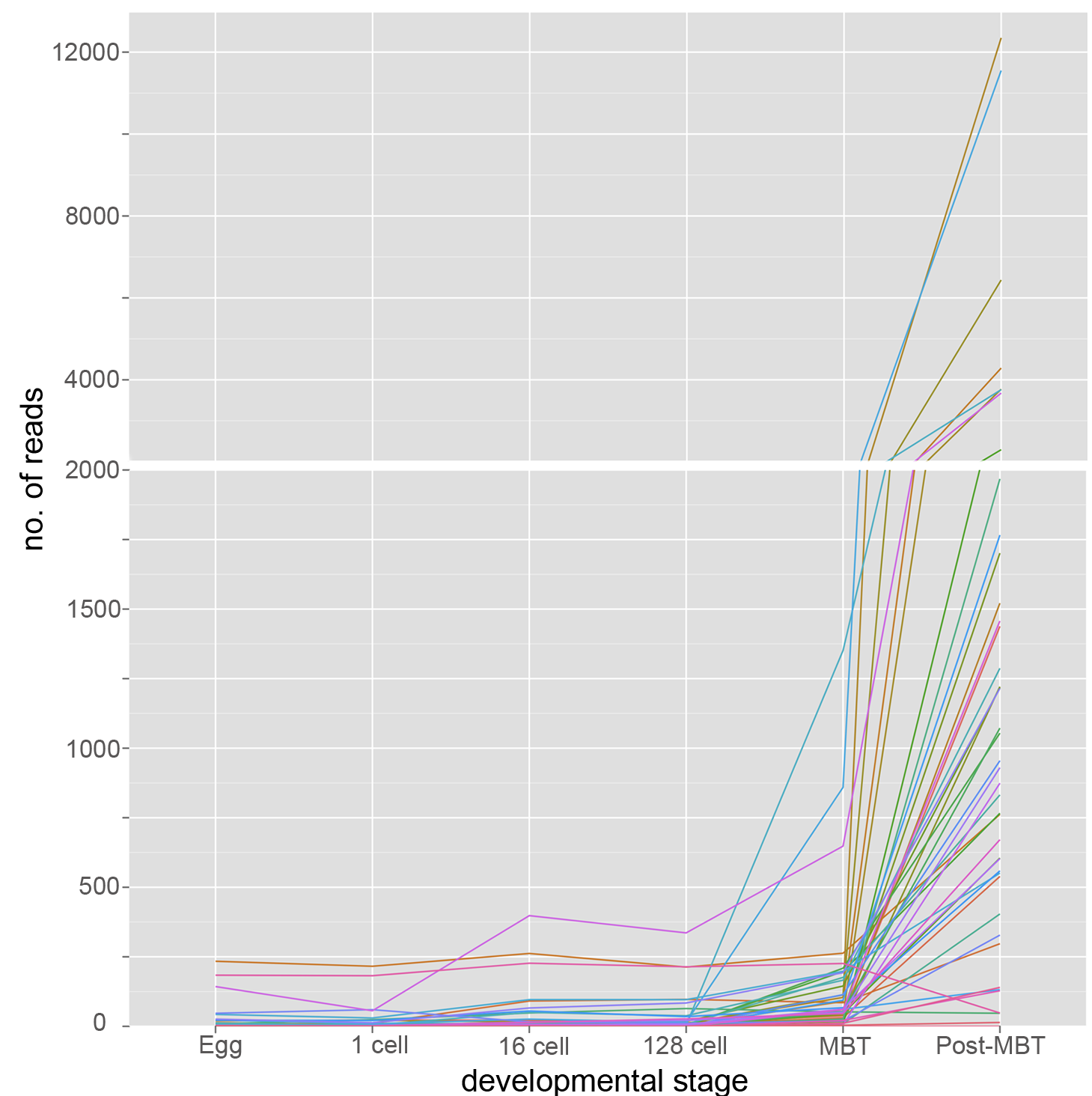

Supplement: Figure S2 — Majority of the candidate genes are upregulated after MBT. Using a previously published dataset ([31] in Main Text), we tested whether the expression of our candidate genes is upregulated at MBT, as expected. Our results show that indeed, this is the case for all, except two genes: map2k6 and tmem68. The former showed a weak maternal expression, which was downregulated after MBT, whereas the latter showed no change in expression during and after MBT. (TIF) [file pone.0070053.s002.tif]

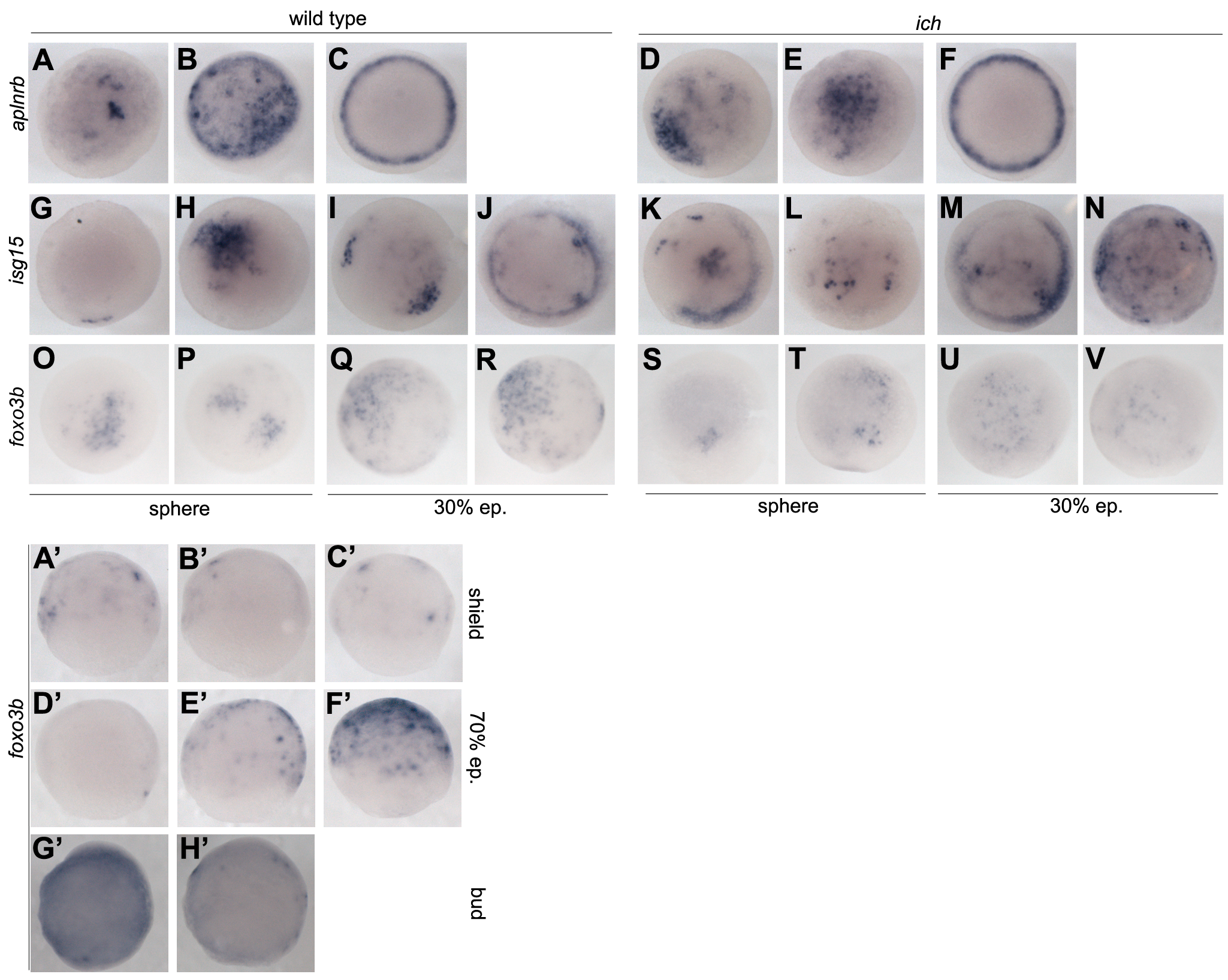

Supplement: Figure S3 — Genes with stochastic early expression. Genes belonging to this class show stochastic expression at the onset of the zygotic transcription. We could detect clusters of cells both in wild type and ich embryos that upregulated these particular genes, but no clear pattern emerged. For aplnrb (A–F) and isg15 (G–N), the expression pattern later became more coherent, and localized around the germring. Interestingly, although no clear expression pattern emerged, foxo3b expression in general appeared higher in wild type embryos (O–V). Later stages of foxo3b expression in wild type embryos also showed stochastic and varying expression patterns (A’–H’). All sphere and 30% epiboly stage embryos are shown from an animal view. Shield, 70% epiboly and bud stage embryos are shown from a lateral view, with dorsal to the right. (TIF) [file pone.0070053.s003.tif]

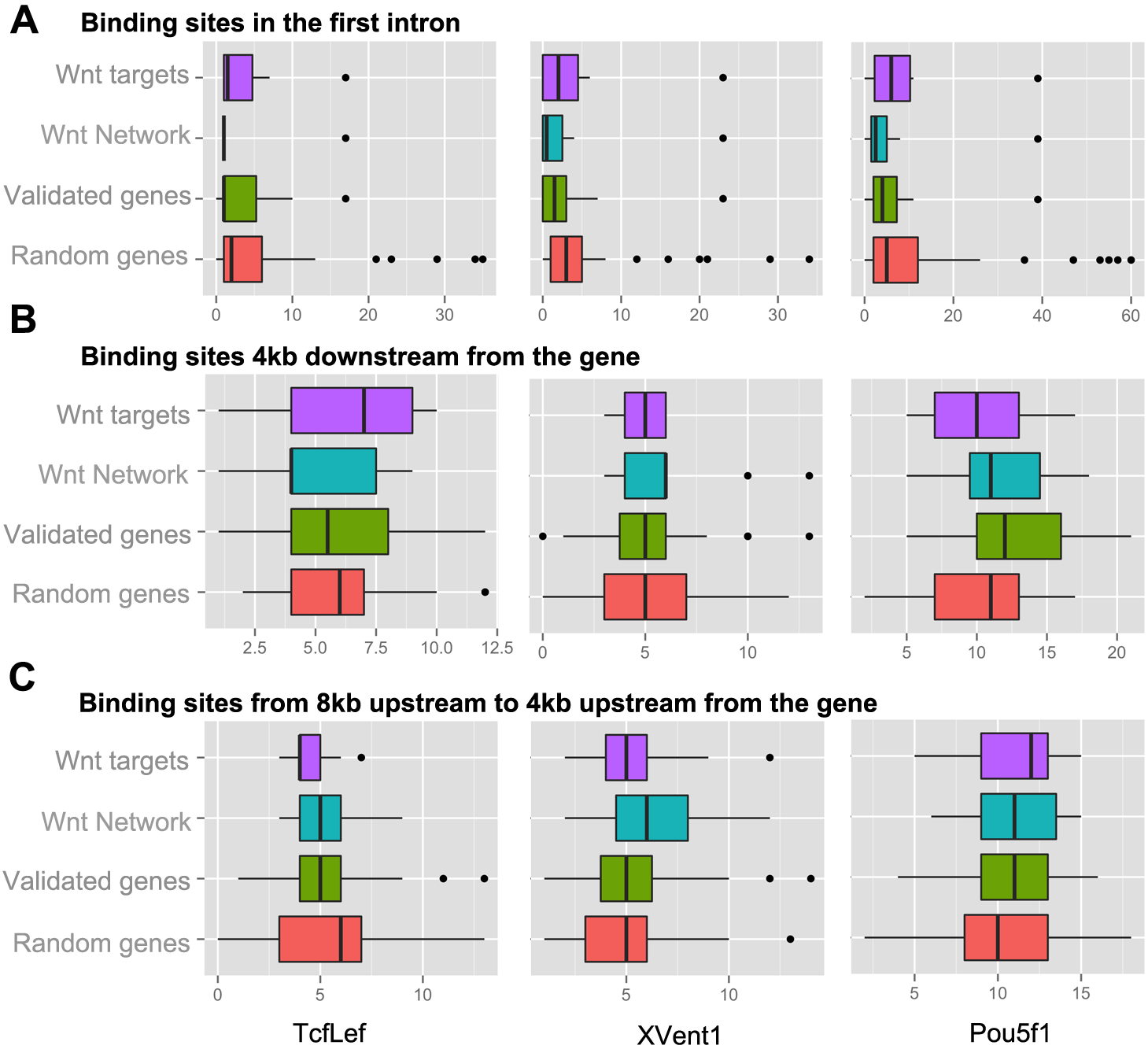

Supplement: Figure S4 — Extended genomic region analysis of validated genes. (A) For multi-exon genes, statistical analysis of the first introns for enrichment in putative TcfLef, XVent1 or Pou5f1 binding sites yielded negative results. (B) In the 4 kb downstream genomic regions, we could not detect significant differences between the random and validated gene-sets in the number of TcfLef and XVent1 sites. The validated gene-set was enriched however in putative Pou5f1 sites (p<0.05). However, this result has to be interpreted with caution, as proven Pou5f1-targets [44] within the validated set have about the same number of potential Pou5f1 binding sites as non-target counterparts (not shown). (C) Analysing the −8 kb to −4 kb upstream genomc region no differences were observed in the number of TcfLef, XVent1 and Pou5f1 sites between our validated and random data-sets. (TIF) [file pone.0070053.s004.tif]
