# Supplementary material for: Full Transcriptome Analysis of Early Dorsoventral Patterning in Zebrafish
Source: PLoS One. 2013 Jul 29;8(7):e70053. doi: 10.1371/journal.pone.0070053 (PMC3726443; doi:10.1371/journal.pone.0070053)
Supplement: Methods S1 — Sequence of PCR primers used for the nested PCR reactions. (DOC) [file pone.0070053.s006.doc]

**SUPPLEMENTARY METHODS**

The primers used for the nested PCR reactions to clone the respective gene fragments for *in situ* hybridization experiments are as follows:

| **Primer name** | **Sequence** |
| --- | --- |
| **arl4ab_Fi** | TCCCCACGTTCGCTTTCATC |
| **arl4ab_Fo** | AACGCTTGGAGGAAGGATCG |
| **arl4ab_Ri** | GCCATCACAGAGGCCTACC |
| **arl4ab_Ro** | AGTCCATGGCAGCTTTGGTA |
| **arrdc3b-Fi** | AAACAGCCGGGGTTTTTATT |
| **arrdc3b-Fo** | GAACAGCAGGCAACTATAGCAA |
| **arrdc3b-Ri** | TCTCCTCATGGAGGAAGAACA |
| **arrdc3b-Ro** | CCAGATTGGGAGTGTTTCCA |
| **clec19a-Fi** | TGGAGAAGGTCTGGATCTCGATGCT |
| **clec19a-Fo** | TGCATGGGCATTGCAGCAGA |
| **clec19a-Ri** | TCCAACAACGGTTTGCGGTCCA |
| **dact2_Fi** | AAAGCATCCACCGAACACCT |
| **dact2_Fo** | CGTCTACGCAGACAGGATGT |
| **dact2_Ri** | CCGACCTTGCCTGCAGTATT |
| **dact2_Ro** | TGTGCATGGACAAACTCGGA |
| **dnase1l3_Fi** | AGAAGCAGAGCAGAATTGTGGA |
| **dnase1l3_Fo** | GTGACTTCAGGCACTCTGGG |
| **dnase1l3_Ri** | TTCAGTCACTCTCCAACGCA |
| **dnase1l3_Ro** | GGATGACTTTCTGACTGGGCA |
| **foxo3b_Fi** | GGCTCTGACAGGTCAGTTCTT |
| **foxo3b_Fo** | GATCACTGCAGCTAACGAGC |
| **foxo3b_Ri** | CCACGTGCAGTCTTGGTGTA |
| **foxo3b_Ro** | CAGTCCAGCACGATTCCTGT |
| **fzd8b-Fo** | CCAGAGCACATGCCAGCGCA |
| **fzd8b-Ro** | GCTGCAGTTGCTCAGTTTGCAGT |
| **hnf1ba-Fi** | CGCATTTCCCTCTCTAGACCTC |
| **hnf1ba-Fo** | GAGGAGCTCACACTTGGAGG |
| **hnf1ba-Ri** | GGCTGGTTGACATGGCATTG |
| **hnf1ba-Ro** | TCAGGGTGCTTACTGGAGGA |
| **ier2_Fi** | GGGGACTGAGGCTTCACCGGA |
| **ier2_Fo** | CATTAAGGACCCAGCGCGGGG |
| **ier2_Ri** | CGCACGGGACGACCTGTTCC |
| **ier2_Ro** | CGTTCAGGAGCGCACCGGTT |
| **isg15-Fi** | AAGTGAGCGGTGATGCTACC |
| **isg15-Fo** | TAAAACTGCTTGGCGGTGAT |
| **isg15-Ri** | TCATAGTCCTGCAACTTCATGC |
| **isg15-Ro** | TGGTGCTCAATGAGGTGATG |
| **LOC100334443_Fi** | GCAGCTTCCGCGTTCCTCCC |
| **LOC100334443_Fo** | TGCCACACAGAAGCAGCGTG |
| **LOC100334443_Ri** | GGGCGCCTTGGGAAAAGGACA |
| **LOC100334443_Ro** | CTGTGCGGCCGGTCTTCGTT |
| **map2k6-Fi** | GGGCTTGTATTTTGCACCTT |
| **map2k6-Fo** | CCTTTGCACGGAGAACAAGT |
| **map2k6-Ri** | TCTTGCGATTGATGTTTTCG |
| **map2k6-Ro** | AGGCGATGCTCACTCATACA |
| **mst1_Fi** | CAGCTCTCCTGCTTCGACTCGA |
| **mst1_Fo** | GCTGTGTGGCTGCAGTAAAGCG |
| **mst1_Ri** | AGATGTTGGGTTGTCCTGCGTG |
| **mst1_Ro** | TCCACATAAACCGAGACGCGGA |
| **notum3_Fi** | CTGCCGTGTTGACTCAGTGT |
| **notum3_Fo** | GCTACAGCGCAACACAAGAG |
| **notum3_Ri** | GCCGCTTGATGGTAGGATAA |
| **notum3_Ro** | CAGGAGTTTACTCGGGTCCA |
| **ppp1r3ca-Fi** | GCATTGTGCTTTGTGCAAGT |
| **ppp1r3ca-Fo** | AGCAGACAGGCTGCAGGT |
| **ppp1r3ca-Ri** | CCTGCAAAGTAATCAGCAGGTA |
| **ppp1r3ca-Ro** | GACCATTTCAACCAGCCACT |
| **rasl11b_Fi** | TTGCTTTTACGGCGTCTGTG |
| **rasl11b_Fo** | ACCAAGTAGAAGTCTTGGTGAGA |
| **rasl11b_Ri** | TGCTCTGGAGATCTGCTCCT |
| **rasl11b_Ro** | ACAGCAACAAAGGCGTTCAC |
| **rnd1l_Fi** | TGCAAACTGGTGCTGGTTGGGG |
| **rnd1l_Fo** | CGCACAGCCACTGGTGGTCA |
| **rnd1l_Ri** | AACCAGCCCTTCCGCAACCG |
| **rnd1l_Ro** | CATCTGGGACGCTCGCGTGG |
| **tmem68-Fi** | CCACAGGAGGAGTGTGTGAA |
| **tmem68-Fo** | TGTTGGAGGTGTTCAGTGTGA |
| **tmem68-Ri** | CGCAGGATGTTTCCAGGTAT |
| **tmem68-Ro** | TTGGAATCGCTCCAAAAGAG |
